# Case of Complete Rupture of the Perineum; Operation; Cure

**Published:** 1857-03

**Authors:** B. F. Trabue

**Affiliations:** Glasgow, Kentucky


ART. V.—*Case of Complete Rupture of the Perineum, of Eighteen Years' Standing; Operation; Cure.* By B. F. TRABUE, M.D., of Glasgow, Kentucky.

IN the spring of 1856, I was called to see Mrs. E. L. K——, a married woman, æt. 35 years, who complained of distressing pain in the region of the bladder, and stated that she had suffered in this way for many years. Finding that I could gain no satisfactory information of the nature of the case by simply questioning the patient, I requested an examination, which being granted, a complete laceration of the perineum, involving both the external and internal sphincters, was revealed. Upon further inquiries, I learned that the accident had happened when she was but 17 years old, when she was confined with her first child, a midwife being in attendance. I explained to her, as well as I could, the actual condition of the parts, of which she seemed to possess a very imperfect idea; and proposed an operation as the only means of relief. To this she consented, confessing that the past eighteen years of her life had been a burden to her, and that without a prospect of improvement, she would much prefer to die. She is the mother of seven living children, and has had three abortions.

On the 13th of May, assisted by Dr. R. H. Grinstead of St. Louis, and Dr. J. B. Thomas, of this place, and in the presence of three ladies, who lent their aid as occasion required, I proceeded to operate. The bowels had been cleared about seven hours before by means of castor oil, and half an hour before the time appointed the rectum had been washed out with an enema of water containing a small quantity of salt.

The patient was placed in the usual position for lithotomy, and well secured to an obstetrical supporter, which is a capital apparatus, and I am not aware was ever before employed for this purpose. Chloroform was not administered, on account of her positive refusal to take it. The first step of the operation consisted in making two flaps, one from each side, about an inch in depth, and an inch and three-fourths in length, extending upward to the margin of the recto-vaginal septum, which latter was then made raw by paring it with the knife. Considerable hemorrhage ensued, but was sufficiently controlled by the application of cold water. I next introduced three double ligatures deep into the parts, entering the needle, each time, at least an inch from the margin of the wound. This was effected by means of a curved needle screwed on to a strong handle, the latter being detached at each introduction, and the former pulled through the parts by means of a pair of long forceps. The three ligatures were then tied over a piece of pipe-stem placed on each side of the wound, as in the usual manner of making the quill-suture. In this way the cut edges were brought in close apposition, but as a further security, I also made use of three superficial interrupted sutures, as advised by Mr. Isaac Baker Brown. Lastly, and also in accordance with the directions of Mr. Brown, I introduced a probe-pointed bistoury about an inch within the bowel, and divided the mucous membrane, skin, and anal sphincter on each side, carrying the knife deep in an outward and backward direction. Without this division of the sphincter, I am satisfied that the operation will seldom succeed.

A small teaspoonful of laudanum having been administered, and the vagina washed out by an injection of cold water, the patient was placed in bed upon her side, with her knees semi-flexed. The time consumed in the entire operation was just one hour.

I visited her six hours subsequently; found her tolerably comfortable; pulse 80; drew off the urine with a catheter; ordered diet consisting of corn-meal gruel, thin squirrel-soup, coffee, and tea; and prescribed 1½ gr. opium to be taken every eight hours.

14th, 9 A.M.; rested well last night; passed urine every eight hours by getting on hands and knees, following each evacuation by an injection of tepid water into the vagina; pulse 80.

The case continued to do well, the patient taking daily a liberal amount of whiskey and water, although heretofore unaccustomed to the use of the former. On the sixth day, I removed the deep sutures, and on the seventh the superficial ones.

On the eighteenth day, I examined the parts carefully, and found that they were firmly united, except at the bottom of the wound, where a small fissure still existed. Ordered ℥ij castor oil, to be followed by enemas of salt and water until free evacuation of the bowels.

During the operation of the medicine, a considerable portion of fecal matter entered the vagina; but the next day the fæces were more consistent, and by pressure made in front the whole passed through the anus.

The fistula subsequently closed without any special treatment, and now, Nov. 1st, the parts are perfectly sound.

It will be observed that for eighteen days this patient was not allowed to empty her bowels, and that no ill consequences resulted from their long confinement.

